# Evaluating the Effectiveness of Diabetes Shared Medical Appointments (SMAs) as Implemented in Five Veterans Affairs Health Systems: a Multi-site Cluster Randomized Pragmatic Trial

**DOI:** 10.1007/s11606-020-06570-y

**Published:** 2021-02-02

**Authors:** Michele Heisler, Jennifer Burgess, Jeffrey Cass, John F. Chardos, Alexander B. Guirguis, Lorrie A. Strohecker, Adam S. Tremblay, Wen-Chih Wu, Donna M. Zulman

**Affiliations:** 1 Veterans Affairs Center for Clinical Management Research, VA Ann Arbor Healthcare System, Ann Arbor, MI USA; 2 Department of Internal Medicine, University of Michigan Medical School, Ann Arbor, MI USA; 3 Department of Health Behavior and Health Education, School of Public Health, University of Michigan, Ann Arbor, MI USA; 4 North Campus Research Complex, University of Michigan, Ann Arbor, MI USA; 5 VA Northern California Health Care System, Mather, CA USA; 6 Veterans Affairs Center for Innovation to Implementation, VA Palo Alto Health Care System, Palo Alto, CA USA; 7 VA Connecticut Healthcare System, West Haven, CT USA; 8 Providence VA Medical Center, Providence, RI USA; 9 Division of General Medicine Disciplines, Stanford University, Stanford, CA USA

**Keywords:** shared medical appointment, peer support, disease management, implementation, diabetes mellitus, pragmatic clinical trial

## Abstract

**Objective:**

To examine whether diabetes shared medical appointments (SMAs) implemented as part of usual clinical practice in diverse health systems are more effective than usual care in improving and sustaining A1c improvements.

**Research Design and Methods:**

A multi-site cluster randomized pragmatic trial examining implementation in clinical practice of diabetes SMAs in five Veterans Affairs (VA) health systems was conducted from 2016 to 2020 among 1537 adults with type 2 diabetes and elevated A1cs. Eligible patients were randomly assigned to either: (1) invitation to participate in a series of SMAs totaling 8–9 h; or (2) continuation of usual care. Relative change in A1c (primary outcome) and in systolic blood pressure, insulin starts, statin starts, and anti-hypertensive medication classes (secondary outcomes) were measured as part of usual clinical care at baseline, at 6 months and at 12 months (~7 months after conclusion of the final SMA in four of five sites). We examined outcomes in three samples of SMA participants: all those scheduled for a SMA, those attending at least one SMA, and those attending at least half of SMAs.

**Results:**

Baseline mean A1c was 9.0%. Participants scheduled for an SMA achieved A1c reductions 0.35% points greater than the control group between baseline and 6-months follow up (*p* = .001). Those who attended at least one SMA achieved reductions 0.42 % points greater (*p* < .001), and those who attended at least half of scheduled SMAs achieved reductions 0.53 % points greater (*p* < .001) than the control group. At 12-month follow-up, the three SMA analysis samples achieved reductions from baseline ranging from 0.16 % points (*p* = 0.12) to 0.29 % points (*p* = .06) greater than the control group.

**Conclusions:**

Diabetes SMAs as implemented in real-life diverse clinical practices improve glycemic control more than usual care immediately after the SMAs, but relative gains are not maintained. Our findings suggest the need for further study of whether a longer term SMA model or other follow-up strategies would sustain relative clinical improvements associated with this intervention.

**Trial Registration:**

ClinicalTrials.gov ID NCT02132676

**Supplementary Information:**

The online version contains supplementary material available at 10.1007/s11606-020-06570-y.

## INTRODUCTION

Health burdens and costs of type 2 diabetes mellitus (T2DM)—a leading cause of morbidity and mortality—continue to soar. One of three US adults without diabetes at age 45 is projected to develop T2DM.^1^ Success of diabetes treatments depends on patients’ initiating and sustaining key behaviors—taking medications, eating healthily, being physically active, self-monitoring. Many patients need self-management support.^2,3^ Health systems thus seek models to improve diabetes self-management support and clinical management that are more low-cost and scalable than offering frequent one-on-one visits with providers.

One potentially effective and efficient model for providing integrated medical care and self-management support is diabetes shared medical appointments (SMAs). SMAs bring groups of patients together with an interdisciplinary team of providers for a series of 60–120-min sessions. Session leaders encourage participants to set behavioral goals and steps to meet these goals (“action planning”),^4^ discuss key areas of diabetes care, and encourage participants to share experiences and self-management strategies with each other.^5,6^ One team provider has prescribing privileges and meets individually with participants to adjust medications.

Some efficacy trials have found SMAs to be more effective than usual care in improving A1c. ^7–11^ In Edelman et al.’s 2010 trial, group medical visits for adults with both high A1cs and blood pressure improved blood pressure but not A1cs.^12^ Meta-analyses have reinforced the efficacy of SMAs,^13–16^ though one found that their heterogeneity does not allow conclusions about which SMA components are necessary for effectiveness.^13^ They emphasized the need now for SMAs to be evaluated as implemented in real-life clinical practice. It is also necessary to examine longer term outcomes after the conclusion of SMAs offered to participants over a limited period, as is the case in most programs. One study examining outcomes 13–18 months after the conclusion of the 2010 Edelman et al. trial found that blood pressure gains relative to the control group were not maintained, but participants had lower rates of hospital inpatient admissions and expenditures. ^17^ However, most studies to date have examined outcomes immediately after participation in SMAs. More research is needed to understand if benefits are sustained after participants return to usual care.

Novel approaches such as mobilizing peer support among SMA participants may help patients sustain improvements achieved through SMAs. Efficacy trials have found that reciprocal peer support programs involving telephone calls between paired participants and periodic peer-led group sessions improve glycemic control more than nurse care management.^18^ It is not known whether patients in real-life SMAs would be interested in participating in such a program and, if so, whether participation would sustain improvements better than a return to usual care alone. Again, whether what is effective in controlled clinical trials led by well-trained research staff is equally effective when implemented in usual clinical practice is unknown.

To address these gaps in knowledge, we worked with five geographically diverse VA health systems implementing diabetes SMAs as part of clinical practice to establish a core set of SMA elements and to develop a rigorous evaluation of these SMAs’ effectiveness. We also sought to examine whether SMA participants offered the opportunity at their first SMA session to pair up with another participant to support each other would sustain improved clinical outcomes more than participants in SMA cohorts not offered this option. We thus conducted a multi-site cluster randomized trial to examine effectiveness of SMAs as implemented in diverse real-life VA settings in improving glycemic control (primary outcomes) and systolic blood pressure (SBP), insulin starts, statin starts, and anti-hypertensive medication class changes (secondary outcomes) compared with usual care. We also examined the feasibility and effectiveness of offering a reciprocal peer support program (P2P) as a complement to participation in the SMAs.

## RESEARCH DESIGN AND METHODS

### Setting

Since 2005, the VA has mandated SMAs, yet had not evaluated their effectiveness as implemented compared with usual care. For this evaluation, we enlisted the support of clinical leadership at five VA health care systems implementing diabetes SMAs (Table 1).

**Table 1:** Health Care Facility and Shared Medical Appointment (SMA) Information

	Ann Arbor	Palo Alto*	Providence	Sacramento	West Haven
Fiscal year 2016: number^‡^ of type 2 diabetes seen in ambulatory care with eligible A1c	11,671	14,545	9017	9407	9367
Shared medical appointment (SMA) clinician team trained in motivational interviewing-based group facilitation and action planning^18 19^	Doctor of Pharmacy (PharmD) Registered Dietician (RD) Registered Nurse (RN) Medical Doctor (MD)^†^ Licensed Practical Nurse (LPN)^†^	PharmD Health Behavior Coordinator (HBC) RD RN MD^†^ Social Worker (SW)^†^ Registered Nurse Practitioner (RNP)^†^ Physical Therapist (PT)^†^	PharmD HBC MD RD RN	MD PharmD HBC RD	PharmD HBC RD RN MD†
Frequency of SMAs/cohort	Monthly	Biweekly	Biweekly	Weekly	Quarterly
# of SMAs/cohort	4	4	6	8	4
Duration of each SMA	2 h	2 h	1.5 h	1 h	2 h
Total SMA dose/cohort	8 h	8 h	9 h	8 h	8 h
Mean SMA cohort size	7.15	8.69	8.85	6.15	5.08
Range of SMA cohort size	5–10	5–13	5–11	3–19	2–10
All sessions include (1) review of participants’ vitals and labs; (2) action planning (goal setting) and discussion of each participant’s progress, challenges, strategies to meet action step and formulation of new step; and (3) prescriber holds brief individual sessions with each participant to review medications and make medication changes as necessary.^‡^	Yes	Yes	Yes	Yes	Yes
Group facilitation focused on creating patient-driven, interactive discussion among participants‡	Yes	Yes	Yes	Yes	Yes
Information and education on medications, blood pressure and lipid control, diet, exercise, stress management that is driven by participants' interests and questions‡	Yes	Yes	Yes	Yes	Yes

*Includes Palo Alto, Livermore, Fremont, and San Jose

†Occasional participant/guest speaker

‡To assess fidelity of SMA sessions across all sites, a trained research staff member attended all SMA sessions for a subset of cohorts at each site and completed a fidelity checklist

### Patient Selection, Recruitment, and Randomization

The study protocol is described elsewhere.^19^ Briefly, from May 2016 to May 2018, we identified from electronic health records (EHR) patients who had (1) two outpatient visits or one hospitalization with a diabetes-related ICD-10 code in the prior 12 months; or (2) at least one prescription for a glucose control medication; and (3) an A1c ≥ 7.5% if age < 70 or ≥ 8.0% if age 70+ years within 6 months prior to enrollment. We excluded patients with active substance abuse disorders, schizophrenia, severe dementia, or bipolar disorder.

As both SMAs and P2P were clinical program offerings, analysis of clinical outcomes among participants did not require Central VA Institutional Review Board (CIRB) oversight. The CIRB granted a waiver of signed consent to allow us to evaluate clinical outcomes from the EHR for all participants.

Patients were identified to be randomized through quarterly data pulls from VA clinical databases. As there were more eligible participants at each site than capacity, patients identified through the EHR each quarter (and also through provider referrals at one site) were randomized by random number generator to usual care or to be invited to participate in SMAs. Some SMA cohorts were then randomly selected for participants to be offered at their first SMA session the opportunity to participate in P2P.

### Usual Care

The study team had no contact with the group who received standard VA services. Eligible patients randomized to usual care were distributed equally across the recruitment period.

### SMA-Only Program

As the aim was to evaluate real-world effectiveness, the sites were given latitude in their SMAs’ frequency, duration, and content. All sites, however, agreed to key shared SMA elements (see Table 1). These elements were selected based on study team expertise, the literature on components found in effective SMAs, and hypothesized mechanisms for effectiveness.^20–25^

### SMA + Reciprocal Peer Support Program

The P2P program was offered at the first SMA session to some SMA groups randomized to SMA+P2P. Participants who agreed to participate were matched with another SMA participant and encouraged to make weekly phone calls with each other between SMA sessions and after completion of the SMA series. They also were offered peer facilitator-led group sessions focused on participants’ diabetes self-management goals every 4–6 weeks for a minimum of 12 months following enrollment in SMAs.

### Data Collection and Outcomes Measures

Our primary outcome was the difference between mean baseline A1c values in 6-month windows preceding baseline and following the 6- and 12-month post-enrollment evaluation periods. Secondary outcomes included change in systolic blood pressure (SBP), anti-hypertensive medication use, statin use, and insulin starts across the same evaluation periods. We hypothesized that SMA participants would be more likely to start insulin and a statin and to have changes in anti-hypertensive medications than those in usual care.

We obtained A1c and SBP values entered in the EHR closest to each timepoint. Data on medication use were obtained through the VHA Corporate Data Warehouse. The 6-month evaluation period was ≥ 3 to < 9 months post-enrollment and the 12-month evaluation period was ≥ 9 to <15 months post-enrollment.

### Sample Size Power Calculations

We estimated we could conduct 10 to 16 SMA groups per site with 8–14 people per group. At a minimum, this would equate to 560 people in active treatment and an equal number of controls. In this scenario, we could detect a 0.5% difference in A1c and a 5-mmHg decline in SBP between groups.

### Analyses

Our primary analysis was an intention to treat analysis (ITT) comparing all eligible participants randomized to be scheduled for a SMA (regardless of whether they attended a SMA) or to usual care. We also conducted analyses comparing outcomes between the usual care group and (1) the attendee group, defined as patients who attended at least one SMA, and (2) the engagement group, defined as patients who attended at least half of offered SMAs.

We conducted a “difference-in-differences” (DID) analysis of A1c and SBP changes using a multilevel linear mixed effect model. ^26,27^ The model included a treatment indicator, a time indicator, the treatment-time interaction (DID estimator), and baseline A1c, age, gender, and race. Random intercepts were included for patients (level-1) nested within site (level-2). In analyses of insulin and statin starts, we implemented a multilevel logistic regression model, and for anti-hypertensive medication class changes, we implemented a multilevel Poisson regression model. ^28,29^

As a robustness check, we repeated analyses on a subset of patients matched 1:1 based on propensity scores. ^30^ The propensity score model was developed using a logistic regression on patient demographics. Although our initial analyses used observed data, because we were relying on data available in the EHR, we anticipated more missing values than if this were an efficacy trial. Accordingly, we used logistic regression to model patients' likelihood of having outcome data and defined strata within which outcome values were missing at random. We then stratified patients according to these propensities and randomly sampled from the observed outcome distribution and imputed these values for missing data within each stratum. In sensitivity analyses, we (1) imputed missing data such that any missing A1c measurements at 6 or 12 months would match the value at baseline, ^31^ (2) examined outcomes among a subset of patients with full outcomes data, and (3) used multiple imputation to impute missing A1cs, race, and age. ^32^ We used the Benjamini-Hochberg procedure to control for multiple comparisons. ^33^

Because only 59 patients in the SMA+P2P group chose to participate in P2P, we did not have statistical power to compare outcomes between the SMA-only and SMA+P2P arms. Therefore, we conducted our primary analysis that combined the two SMA groups (SMA-only and SMA+P2P) into one active treatment group and compared clinical outcomes of these patients with those in usual care. In an exploratory analysis, we examined changes in A1c among the 59 P2P participants.

## RESULTS

### Participant Flow and Baseline Data

**Figure 1:**
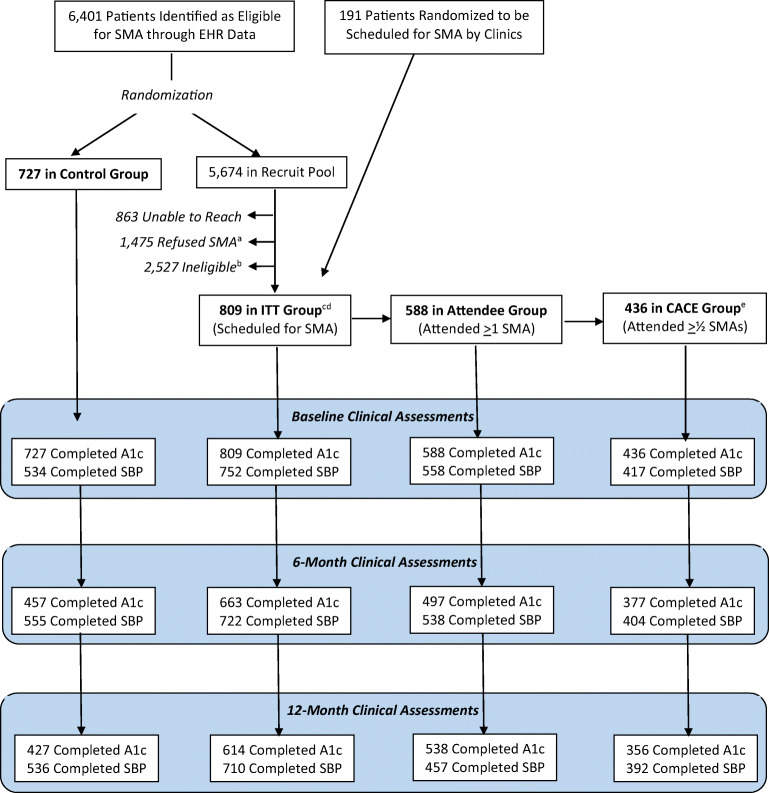
**The CONSORT diagram.**^**a**^**This includes “soft refusers” (e.g., those who did not show up to a scheduled SMA and were unable to be rescheduled).** ^**b**^**This includes those whose A1c’s “expired” (≥ 6 months old) before they could be recruited for an SMA.** ^**c**^**Of these, 304 were offered the P2P program and, of those, 59 actively participated in the P2P program.** ^**d**^**Of these, 451 completed a baseline survey, 376 completed a 6-month survey, and 348 completed a 12-month survey.** ^**e**^**CACE (Complier Average Causal Effect) analysis consists of those meeting our pre-specified threshold for effective engagement.**

The CONSORT diagram (Fig. 1) shows participant flow. Participants were more likely than non-participants to be non-white, have higher levels of education, report poorer health, be less likely to be on insulin, and report less satisfaction with their level of social support. 810 (35% of contacted patients) were scheduled to attend a SMA. Of these, 588 attended at least 1 SMA (73%), and 436 (54%) attended half or more of SMAs, a level of engagement defined a priori as likely to be an adequate dose of participation. (See Appendix 1 for chi-square analyses comparing characteristics between groups and between participants with differing levels of SMA engagement.)

Participants’ baseline characteristics are reported in Table 2. We had complete 6-month outcome data on 457 control (63%) and 663 SMA participants (82%) and complete 12-month data on 427 control (60%) and 614 SMA participants (76%).

**Table 2:** Baseline Characteristics of Intervention and Control Group Patients

Characteristic	Intervention (*N* = 809)	Control (*N* = 727)
	*N* (%) or mean ± SD	
Age (years) as of 6/1/18	67.1 ± 9.2	67.8 ± 12.7
Male	782 (97%)	707 (97%)
Race*		
White	550 (68%)	540 (74%)
Black	150 (19%)	89 (12%)
Asian	29 (4%)	26 (4%)
American Indian/Alaskan Native	8 (1%)	7 (1%)
Hawaiian/Pacific Islander	20 (2%)	11 (2%)
Missing	60 (7%)	61 (8%)
Ethnicity		
Hispanic or Latino	70 (9%)	64 (9%)
Not Hispanic or Latino	702 (87%)	626 (86%)
Missing	37 (5%)	37 (5%)
Most recent hemoglobin A1c in the last 8 months (%)	9.1 ± 1.5	8.9 ± 1.3
Mean systolic blood pressure over the last 8 months (mmHg)	136.2 ± 13.4	137.7 ± 15.2
Most recent systolic blood pressure in the last 8 months (mmHg)	137.2 ± 17.5	139.6 ± 18.9
On insulin	385 (48%)	255 (35%)
On statin	603 (75%)	470 (65%)
Classes of antihypertensive medications	2.4 ± 1.2	2.2 ± 1.3
Primary care in-person visits in past 8 months	3.4 ± 3.1	3.2 ± 3.4
Primary care phone visits in past 8 months	0.7 ± 1.2	0.7 ± 1.5
Nurse Case Manager in-person visits in past 8 months	0.2 ± 0.7	0.2 ± 0.8
Nurse Case Manager phone visits in past 8 months	0.3 ± 0.9	0.3 ± 0.9
Endocrinology in-person visits in past 8 months	0.3 ± 0.8	0.2 ± 0.8

*Patients can have more than 1 race listed, so these do not add to 100%

### Changes in Primary Outcome of A1c

SMA participants achieved clinically and statistically significant greater reductions in A1c than those in the control group between baseline and 6 months, but differences between groups were no longer statistically significant at 12 months (Table 3). Participants scheduled for a SMA achieved A1c reductions 0.35% points greater than the control group between baseline and 6-months follow up (*p* = .001). Those who attended at least one SMA session achieved reductions of 0.42% points greater (*p* < .001), and those who attended at least half of scheduled sessions achieved reductions of 0.53% points greater (*p* < .001) than the control group. At 12-month follow-up, the three SMA samples achieved reductions from baseline ranging from 0.16% points (*p* = 0.12) to 0.29% points (*p* = .06) greater than the control group, none of which were significant at the *p* < .05 level. The 59 P2P participants improved in A1c from a mean of 8.89% to 8.15% at 6 months and 8.25% at 12 months. All sensitivity analyses addressing missing data were similar to our main analysis and showed the same dose-response effects (see Appendix 2). Similarly, A1c improvements in the SMA group were unchanged when P2P participants were excluded from analyses.

**Table 3:** Changes in Primary and Secondary Outcomes Between Baseline and 6 Months and Baseline and 12 Months

	Usual care ITT (*N* = 727)		SMA ITT* (*N* = 810)		SMA attendee^†^ (*N* = 588)		SMA engagement^‡^ (*N* = 436)		Between-group 6-month differences (*p* value)			Between-group 12-month differences (*p* value)		
	0- to 6-month change	0- to 12-month change	0- to 6-month change	0- to 12-month change	0- to 6-month change	0- to 12-month change	0- to 6-month change	0- to 12-month change	SMA ITT v usual care ITT	SMA attendee v usual care ITT	SMA engagement v Usual care ITT	SMA ITT v usual care ITT	SMA attendee v usual care ITT	SMA engagement v usual care ITT
Physiologic measures														
A1c (%)	− 0.66 (< .001)^§^	− 0.79 (< .001)	− 1.01 (< .001)	− 0.95 (< .001)	− 1.08 (< .001)	− 1.00 (< .001)	− 1.19 (< .001)	− 1.08 (< .001)	− 0.35 (0.001)	− 0.42 (< 0.001)	− 0.53 (< 0.001)	− 0.16 (0.12)	− 0.21 (0.07)	− 0.29 (0.06)
SBP (mmHg)	0.88	− 0.80	− 0.47	0.63	− 0.80	− 0.42	− 0.41	− 0.85	− 1.35 (0.340)	− 1.88 (0.219)	− 2.05 (0.234)	1.43 (0.316)	0.17 (0.911)	− 0.82 (0.634)
† Med change measures^‖^														
Insulin starts (yes/no)	0.72	0.69	2.17 (< .001)	2.09 (< .001)	2.44 (< .001)	2.86 (< .001)	2.69 (< .001)	3.59 (< .001)	3.00 (0.006)	3.56 (0.003)	4.60 (0.005)	3.02 (0.005)	3.80 (0.002)	5.68 (0.001)
Statin starts (yes/no)	0.81	0.69*	1.35	1.23	1.55	1.51	2.07 (< .001)	1.99 (< .001)	1.67 (0.067)	1.93 (0.034)	2.83 (0.006)	1.79 (0.037)	2.31 (0.007)	2.63 (0.011)
Anti-HTN med class changes	1.02	1.02	1.01	1.01	1.01	1.02	1.01	1.01	0.99 (0.823)	0.97 (0.690)	0.99 (0.839)	0.99 (0.849)	0.99 (0.897)	0.99 (0.854)

*Includes all those scheduled for an SMA

†Includes all those who attended ≥ 1 SMA

‡Includes all those who attended ≥ ½ of SMAs in series

§*p* value of intra-group change from baseline

‖Values for Insulin starts and Statin starts are odds ratios, and values for anti-hypertensive medication class changes are incidence rate ratios

### Secondary Outcomes

There were no significant differences in SBP changes between the control group and any of the three SMA analytical samples at 6 months or 12 months (Table 3). There were significantly more insulin and statin starts in all three SMA samples at 6 months and 12 months than in the control group (Table 3). There were no differences in changes in anti-hypertensive medication classes between groups.

## DISCUSSION

This study contributes to the literature by providing data on the effectiveness of SMAs as implemented as part of normal clinical practice. Across five geographically diverse VA health systems, diabetes patients with high A1cs who were scheduled for a SMA, who attended at least one SMA, and who attended at least half of SMAs all achieved statistically and clinically significant reductions in A1c at six-months follow-up that were greater than patients in usual care. There was a dose response in effects: Patients who attended at least half of scheduled SMAs achieved A1c reductions 0.11 % points greater than patients who attended at least one SMA and 0.18% points greater than those scheduled for a SMA. At 12 months, both intervention and control groups achieved clinically and statistically significant A1c reductions from baseline, with no significant differences between groups. Mean A1c reductions from baseline to 12 months ranged from 0.95 to 1.08% points for patients in the SMA groups. A mean difference in A1c level of 0.5% translates into an absolute 2.8% risk reduction in diabetes events over 10 years, or a number needed to treat of 36 (i.e., of 36 treated, one person with reduction in diabetes events). ^34^

SMA participants maintained more insulin starts and statin starts over the 12-month study period than patients in the control group, with no differences in anti-hypertensive medication class changes. There were no significant reductions in SBP between baseline, 6 months, and 12 months in any group. This is in contrast to the 2010 Edelman et al trial that found improvements in blood pressure but not in A1c at the end of their group medical visits.^12^ However, whereas their participants at baseline had both poor glycemic and blood pressure control, our participants’ baseline blood pressure control was relatively good, with mean baseline SBPs of < 140 mmHg. Thus, they and the SMA facilitators could focus more on efforts to improve glycemic control. Moreover, the sessions in that trial were more didactic, whereas ours were designed to be patient-directed, interactive, incorporating action planning to set behavioral goals at each session, approaches that have been found to contribute to improved glycemic control. ^20,23–25,35^

This evaluation of actual implementation of diabetes SMAs extends prior findings from efficacy trials that found significant reductions in A1c at the conclusion of SMAs compared with usual care. ^7–11,13–16^ In many areas of clinical innovation, programs found to be efficacious in RCTs conducted under well-controlled conditions are not effective when implemented under real-life conditions. ^36–38^ While relative A1c improvements at 6 months follow-up in our evaluation were more modest than improvements in some efficacy trials, they were still sufficiently greater to confer clinical benefit.

Our findings of improved glycemic control at completion of this model of diabetes SMAs are encouraging. Yet, as is the case for many short-term programs, there was no longer a significant difference between SMA and control patients 8–9 months after the end of the SMA series. Relative improvements in A1c at 12 months among participants who attended at least half of SMA sessions approached statistical significance (*p* = .06), but did not achieve it. SMA participants’ greater uptake in statins and insulin was sustained at 12 months, and participation in SMAs may have other benefits, such as greater access to and more appropriate health care use, lower expenditures and hospitalizations over time as found in Jackson et al's follow up study,^17^ and improved patient-centered outcomes. We are exploring some of these outcomes in other research.

An important question is how to sustain the comparative gains achieved through SMAs. ^39^ Our findings suggest that ongoing SMAs may be needed to sustain this intervention’s relative advantages. Trento et al. found continued gains in A1c and other diabetes outcomes over the course of a 5-year group diabetes care model.^8^ We had hypothesized that offering participants at the first SMA the P2P program might be an effective and scalable approach to maintaining gains. Very few SMA participants elected to participate in this option, however. It is possible that uptake would have been greater if the option of P2P had been offered again later in the SMA series once participants had the chance to get to know each other and establish rapport and trust. Anecdotally, a number of SMA participants reported having contact outside of the SMA sessions, but we did not examine this systematically. In any case, the low uptake of P2P suggests that this approach as currently designed—while effective in efficacy trials^18^—did not translate well in real-life implementation in these five health systems. Implementation in usual clinical practice of other promising models, including phone calls, emails, texts, or other modalities of proactive outreach by trained peer coaches or other health care team members, should be rigorously evaluated.

Our study has limitations. First, all study sites, though geographically diverse, were VA healthcare systems, and patients were predominantly white and men. Results may not generalize to other settings and populations. Second, as an implementation study, all clinical outcomes were gathered from EHRs. Accordingly, we had lower rates of follow-up clinical data and larger windows for capture of data than if we were bringing participants in to collect data by a research team. Our sensitivity analyses to address this deficiency, however, found no significant differences in findings.

In conclusion, our evaluation of implementation of diabetes SMAs within routine clinical programming across five healthcare systems demonstrated improved A1Cs soon after the SMA series' conclusion. SMAs did not improve A1c levels compared with usual care at 12 months, and few patients were interested in the supplementary peer support intervention. These findings add to the evidence that short-term SMAs are effective for diabetes, including when implemented as part of routine clinical care. There remains a great need to test longer term interventions and scalable follow-up programs to help sustain relative improvements achieved through short-term programs.

## Supplementary Information

ESM 1 (DOCX 974 kb)

ESM 2 (DOCX 18 kb)
