# Supplementary material for: Evaluating the Effectiveness of Diabetes Shared Medical Appointments (SMAs) as Implemented in Five Veterans Affairs Health Systems: a Multi-site Cluster Randomized Pragmatic Trial
Source: J Gen Intern Med. 2021 Feb 2;36(6):1648–55. doi: 10.1007/s11606-020-06570-y (PMC8175536; doi:10.1007/s11606-020-06570-y)

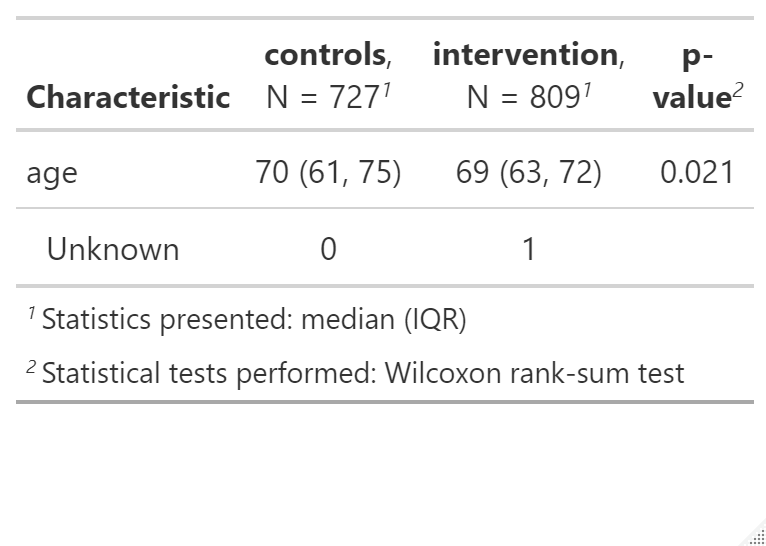


**Appendix 2: Differences between SMA and Control Groups and across Levels of SMA Engagement**


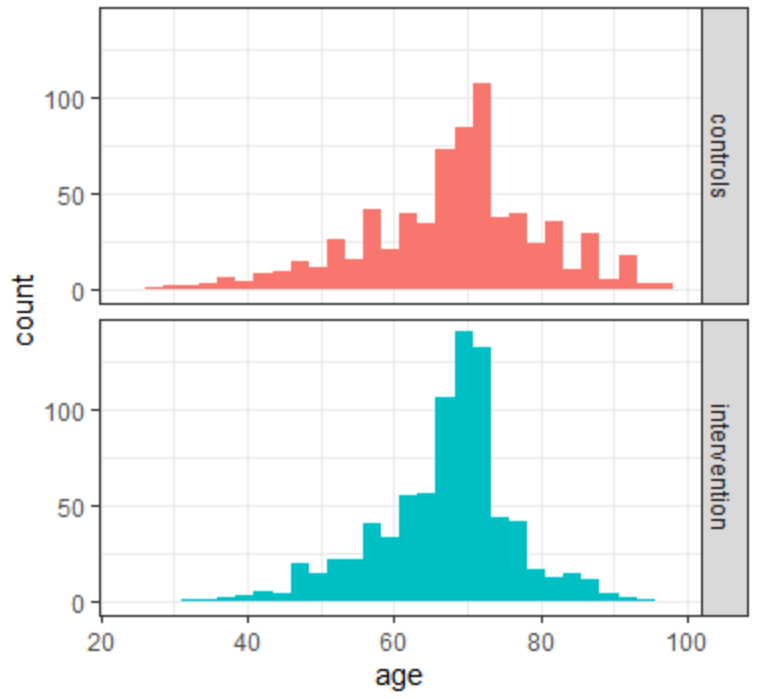


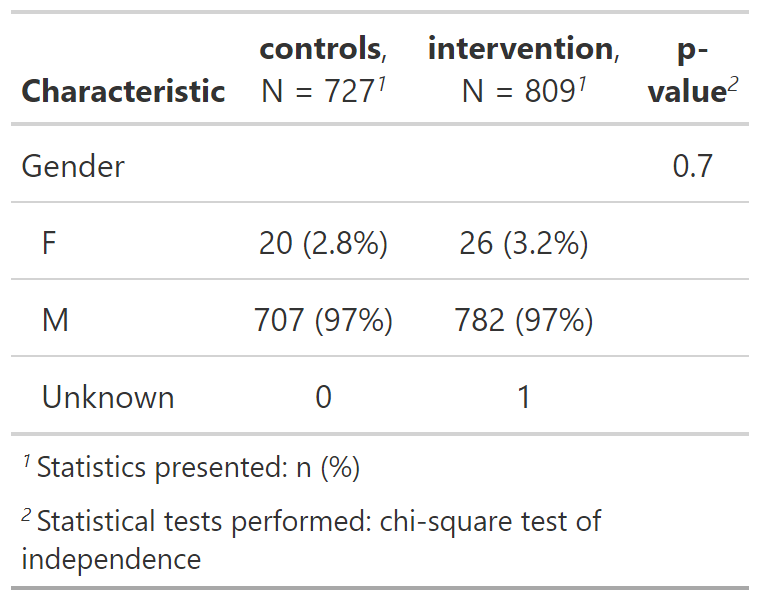


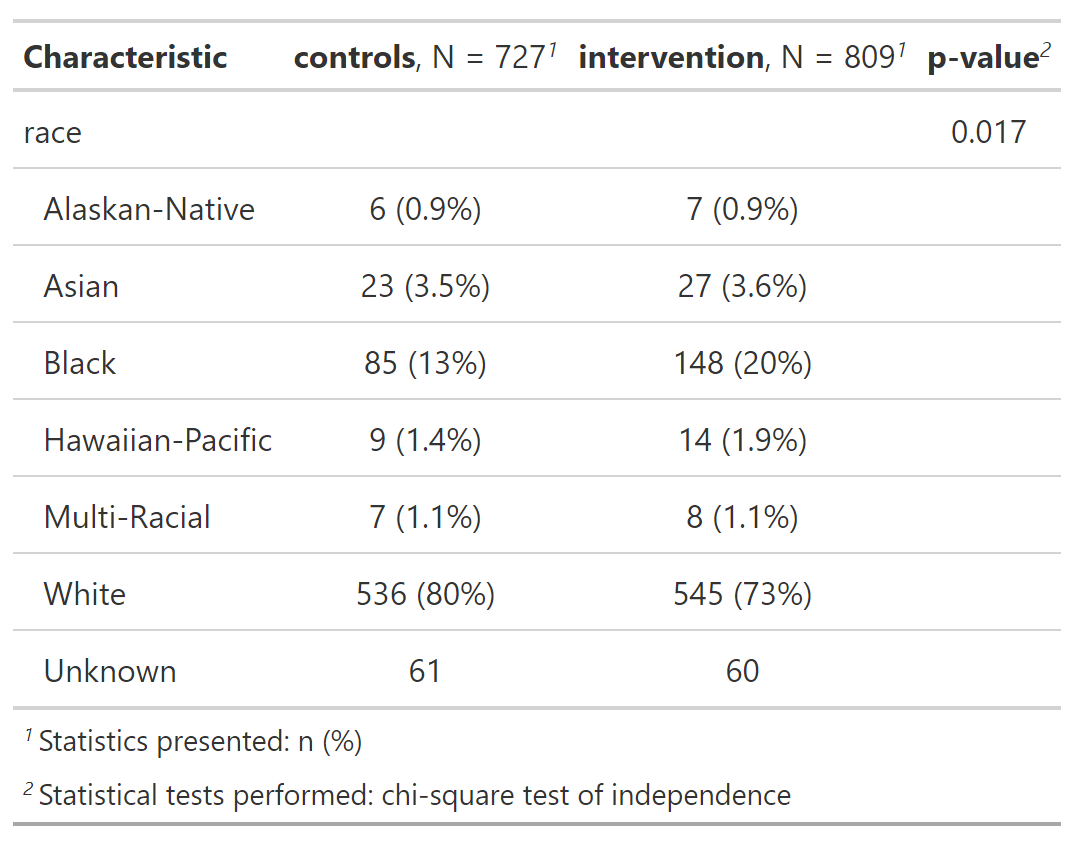


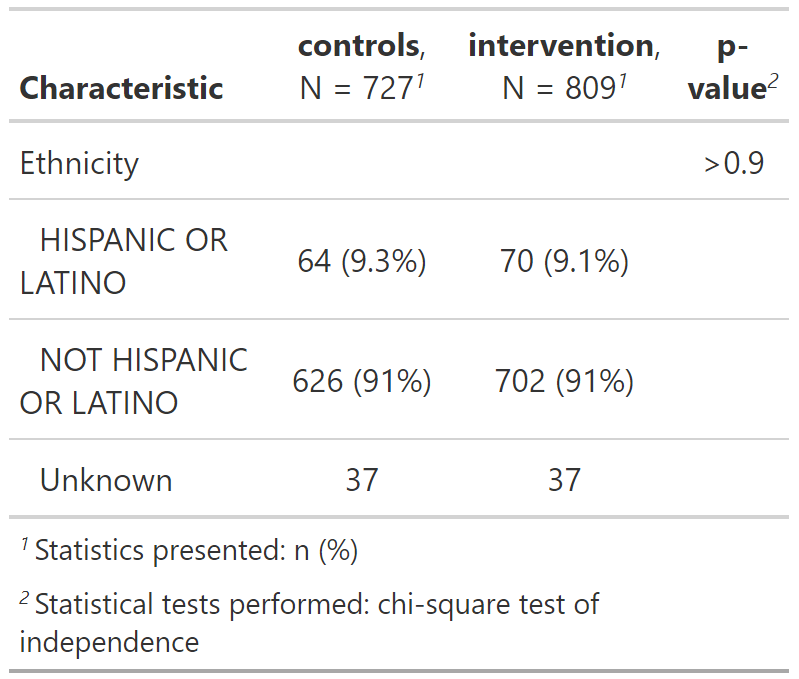


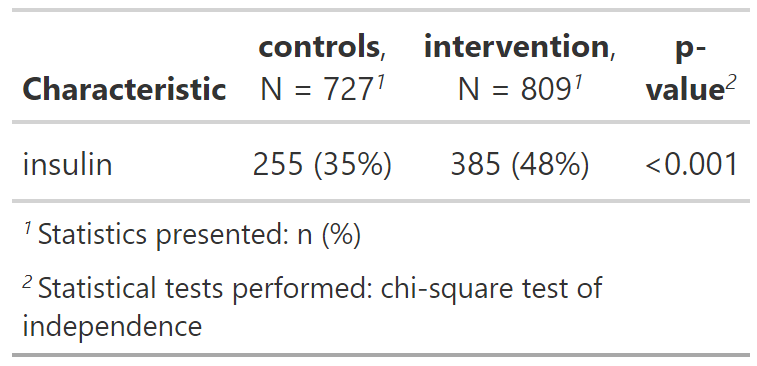


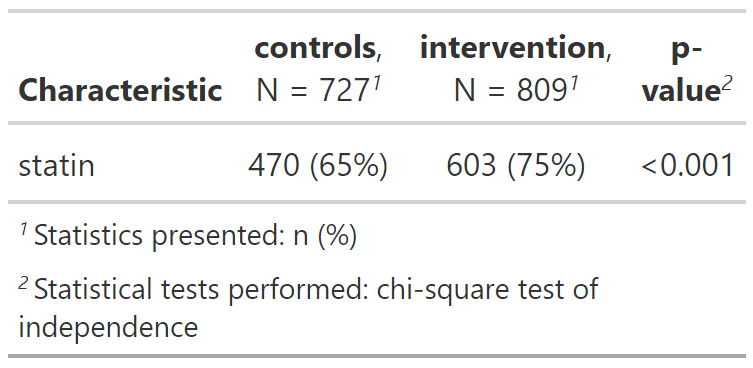


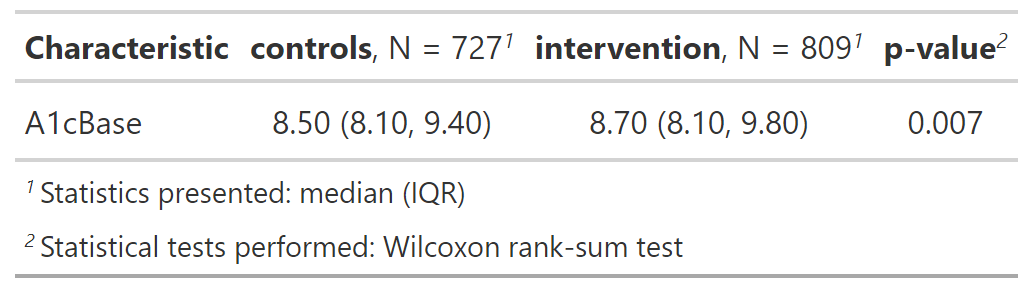


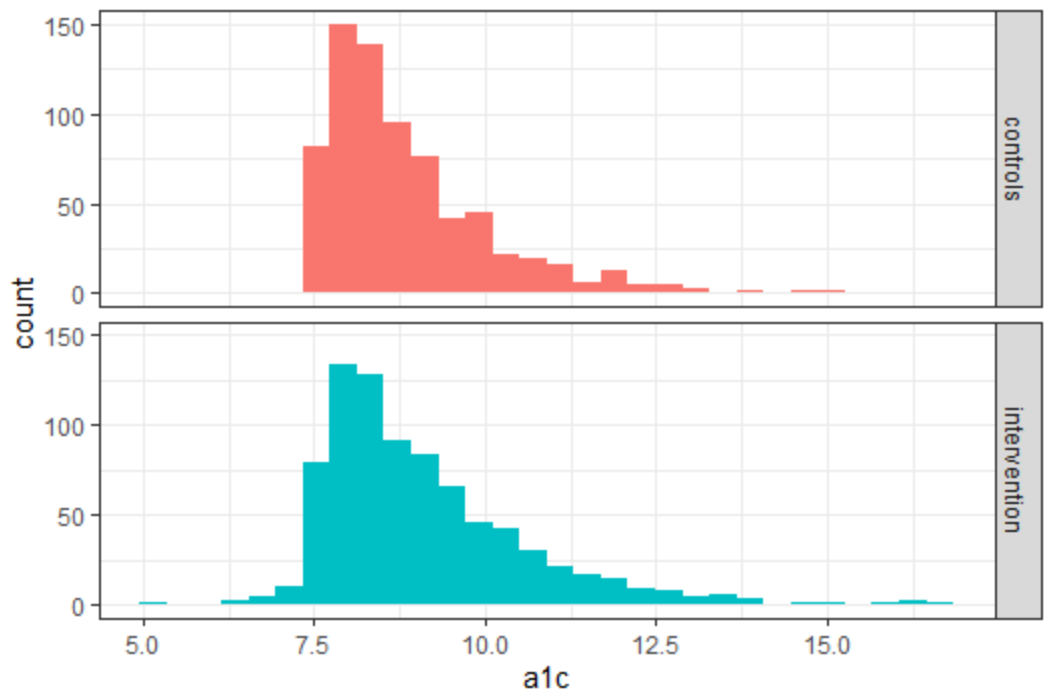


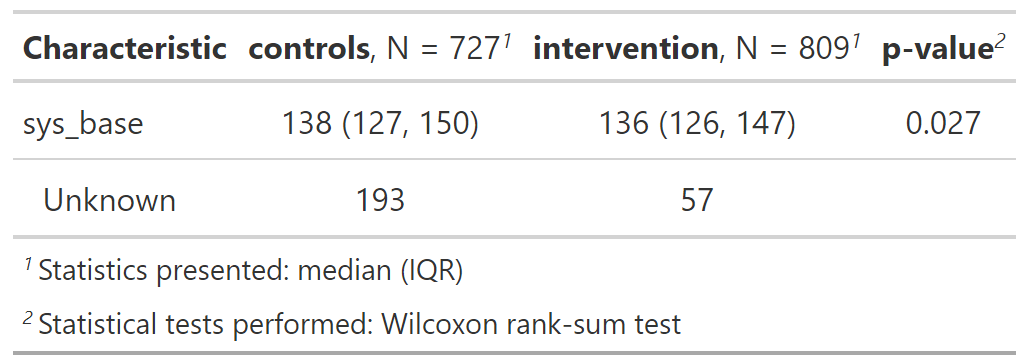


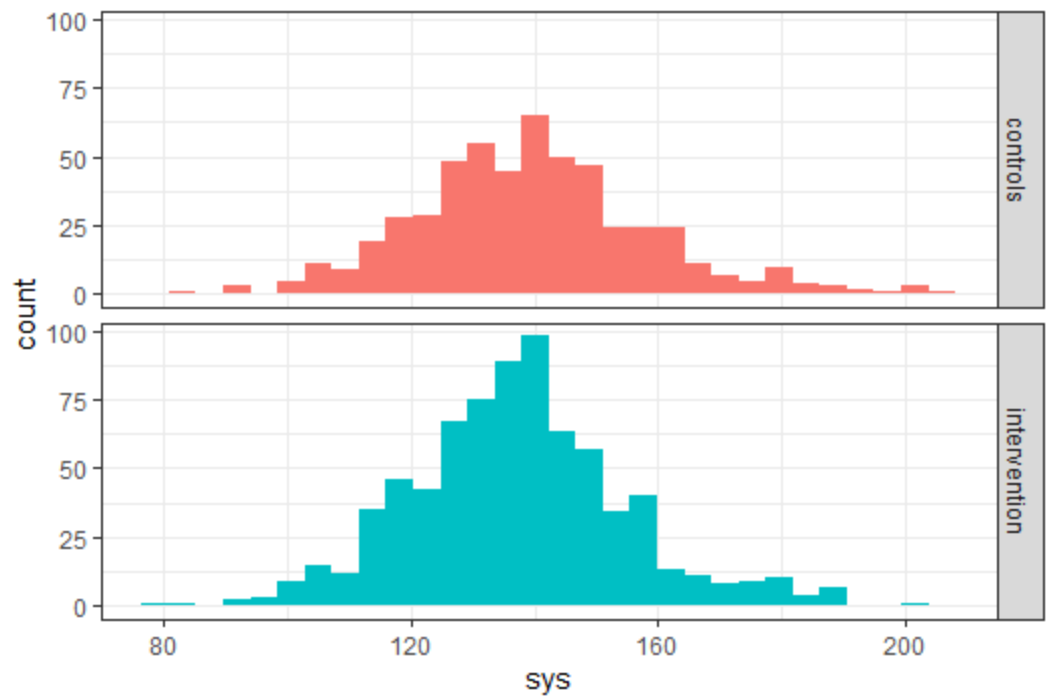


**Among the 809 Intervention patients, they are further split into attendees and non-attendees**


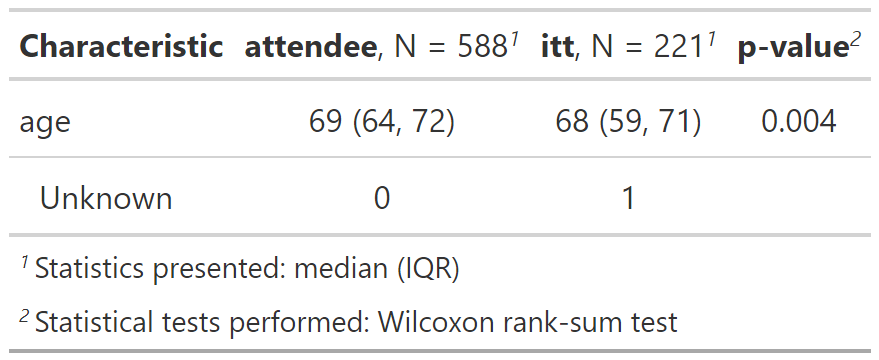


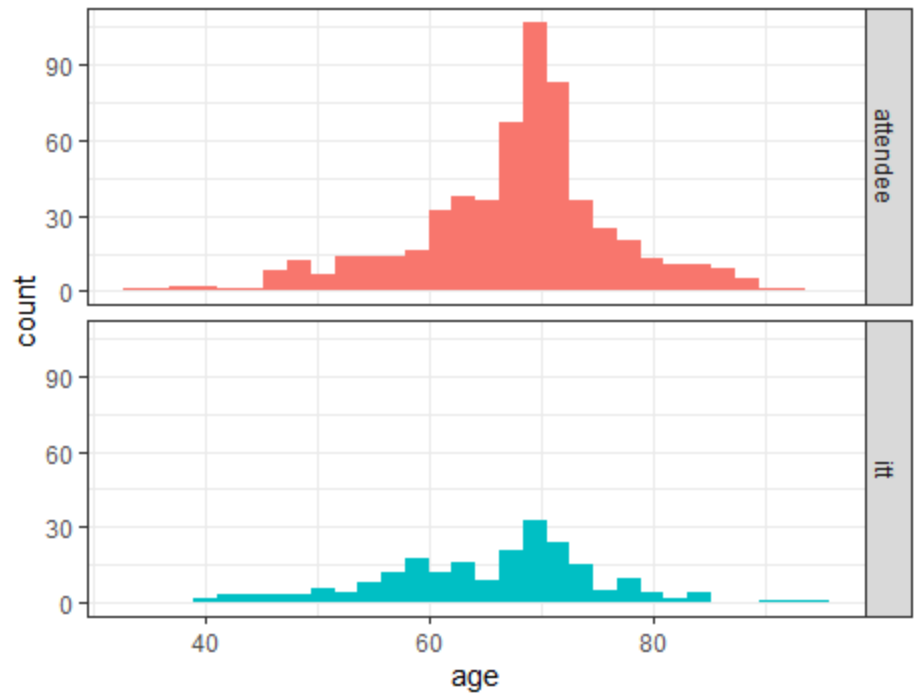


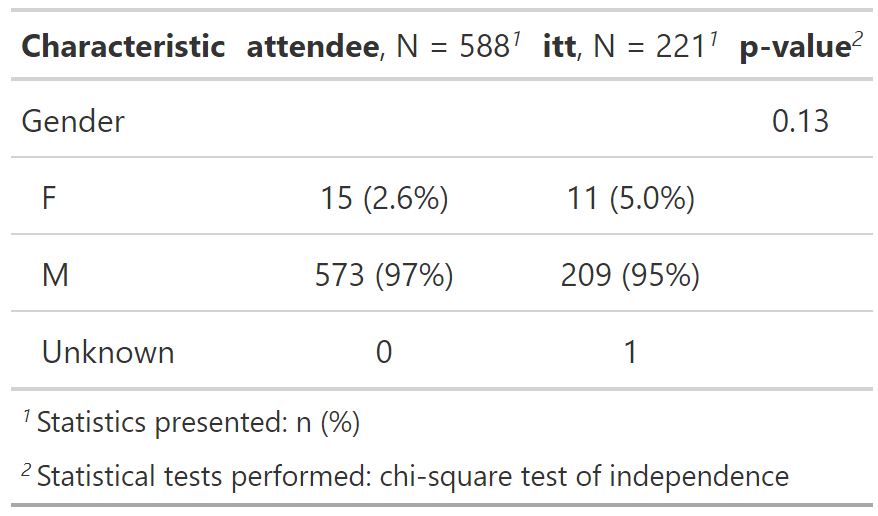


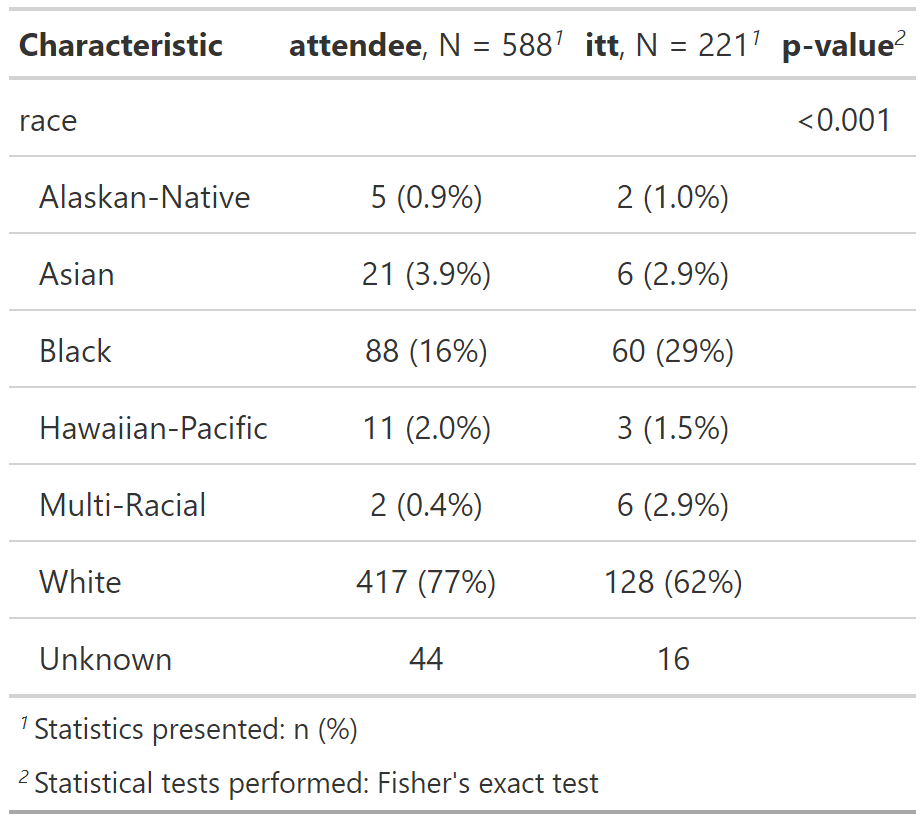


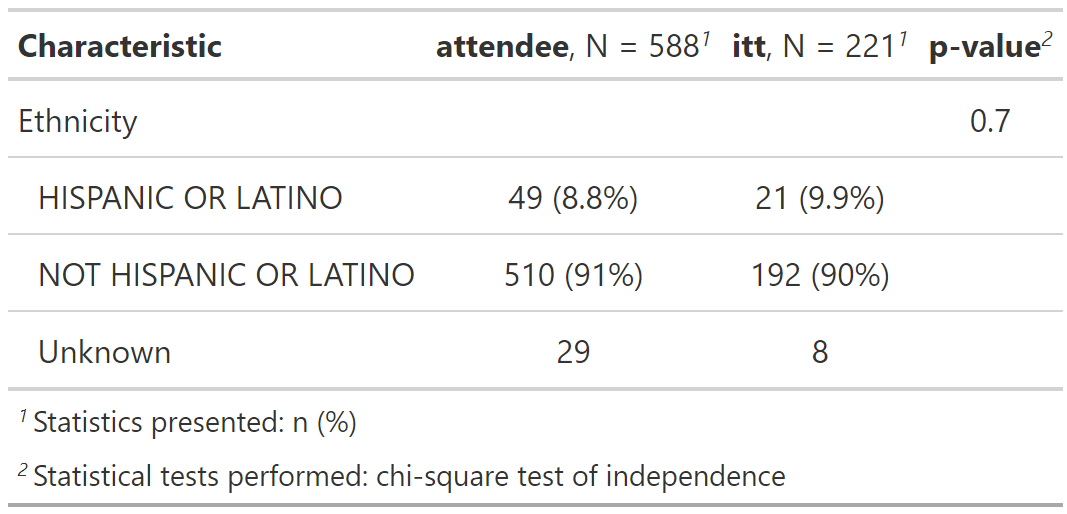


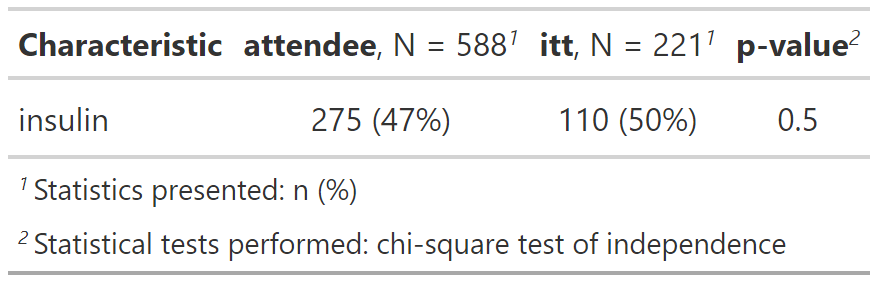


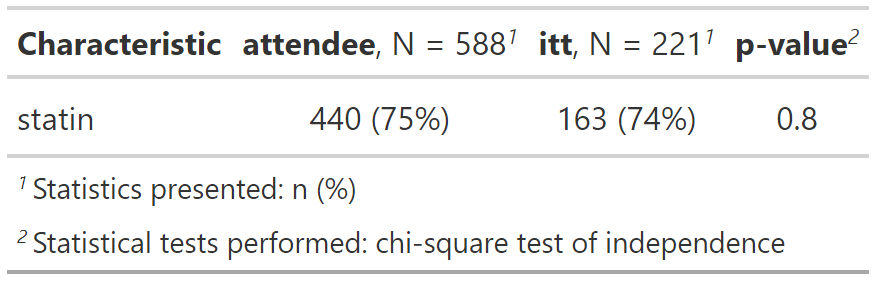


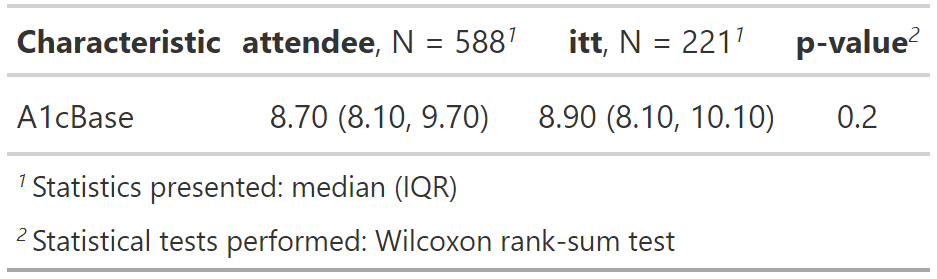


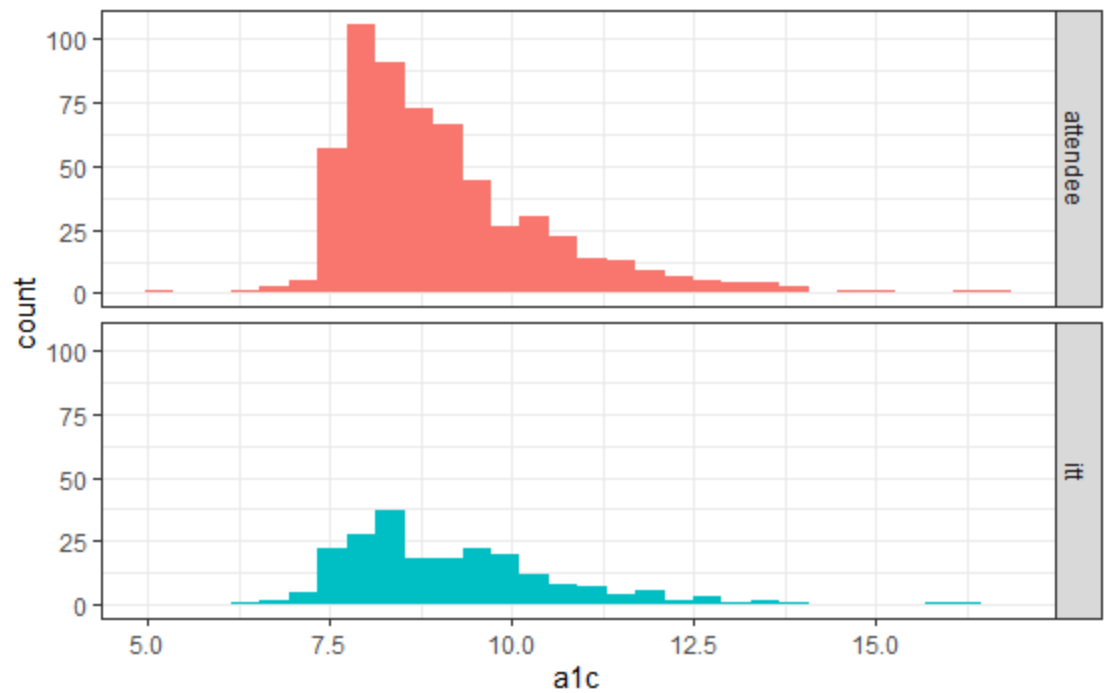


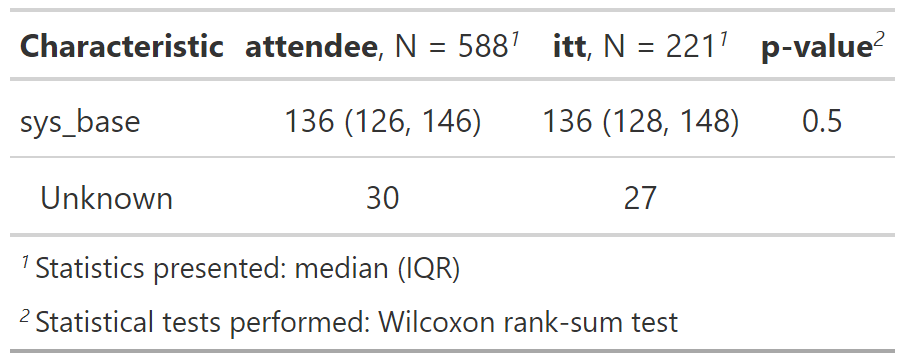


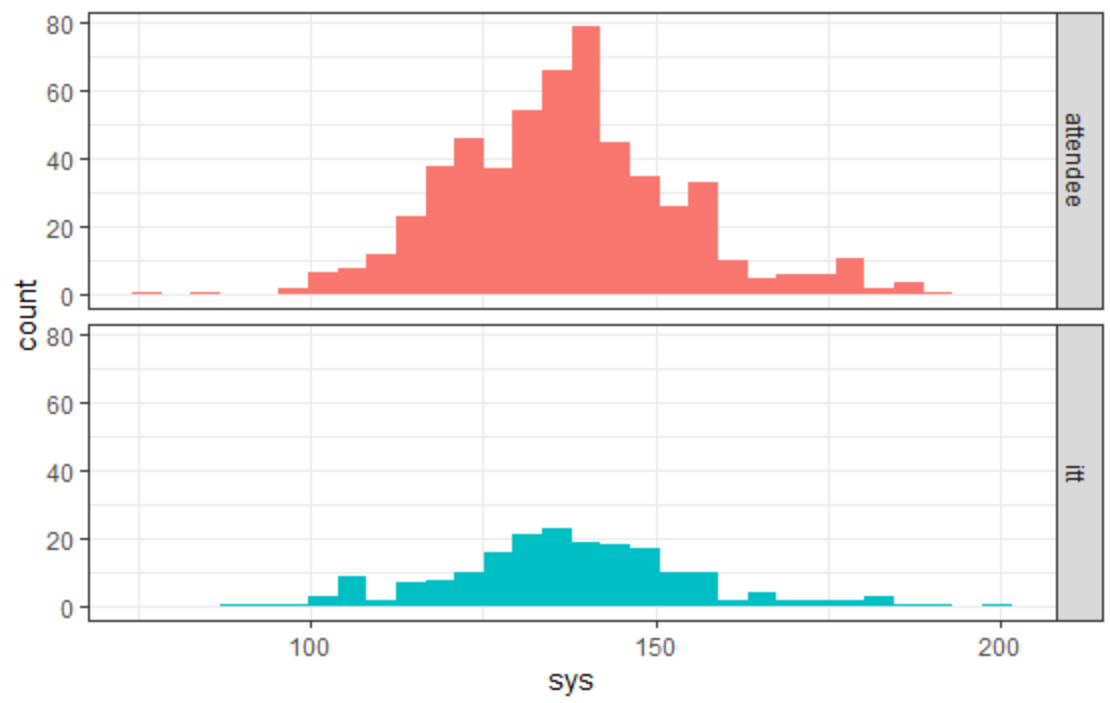

Supplement: Supplementary file 1 — (DOCX 974 kb) [file 11606_2020_6570_MOESM1_ESM.docx]
