# Supplementary material for: Evaluating the Effectiveness of Diabetes Shared Medical Appointments (SMAs) as Implemented in Five Veterans Affairs Health Systems: a Multi-site Cluster Randomized Pragmatic Trial
Source: J Gen Intern Med. 2021 Feb 2;36(6):1648–55. doi: 10.1007/s11606-020-06570-y (PMC8175536; doi:10.1007/s11606-020-06570-y)
Supplement: Supplementary file 2 — (DOCX 18 kb) [file 11606_2020_6570_MOESM2_ESM.docx]

**Appendix 2: Primary Outcomes Results and Sensitivity Analyses**

|  | **Usual Care ITT**  **(N=727)** | | **SMA ITT ^a^**  **(N=809)** | | **SMA Attendee ^b^**  **(N=588)** | | **SMA Engagement ^c^**  **(N=436)** | | **Between-Group 6-Month Differences**  **(p-value)** | | | **Between-Group 12-Month Differences**  **(p-value)** | | |
| --- | --- | --- | --- | --- | --- | --- | --- | --- | --- | --- | --- | --- | --- | --- |
|  | 0 to 6 month change | 0 to 12 month change | 0 to 6 month change | 0 to 12 month change | 0 to 6 month change | 0 to 12 month change | 0 to 6 month change | 0 to 12 month change | SMA ITT  v Usual Care ITT | SMA Attendee  v Usual Care ITT | SMA Engagement v Usual Care ITT | SMA ITT  v Usual Care ITT | SMA Attendee  v Usual Care ITT | SMA Engagement v Usual Care ITT |
| **A1c (%)** | | | | | | | | | | | | | | |
| Main Analysis | -0.66  (<.001)* | -0.79  (<.001) | -1.01  (<.001) | -0.95  (<.001) | -1.08  (<.001) | -1.00  (<.001) | -1.19  (<.001) | -1.08  (<.001) | -0.35 (0.001) | -0.42 (<0.001) | -0.53 (<0.001) | -0.16 (0.12) | -0.21 (0.07) | -0.29  (0.06) |
| Missing Values Match Baseline | -0.43* | -0.51* | -0.79* | -0.70* | -0.85* | -0.73* | -0.97* | -0.79* | -0.36 (<0.001) | -0.42 (<0.001) | -0.55 (<0.001) | -0.22 (0.004) | -0.26 (0.001) | -0.32 (<0.001) |
| Subset of Patients w/ Full Follow-up^1^ | -0.63* | -0.83* | -0.85* | -0.97* | -0.88* | -1.00* | -0.98* | -1.00* | -0.22 (0.048) | -0.24 (0.031) | -0.35 (0.003) | -0.13 (0.232) | -0.15 (0.196) | -0.17 (0.163) |
| Multiple Imputation | -0.64* | -0.78* | -0.90* | -0.94* | -0.93* | -1.00* | -1.02* | -1.01* | -0.25 (0.006) | -0.28 (0.006) | -0.35 (0.002) | -0.17 (0.075) | -0.21 (0.066) | -0.23 (0.025) |

^a^ Includes all those scheduled for an SMA

^b^ Includes all those who attended ≥ 1 SMA

^c^ Includes all those who attended ≥ ½ of SMAs in series

* p-value of intra-group change from baseline

^1^Sample Sizes for Subset of Patients with Full Follow-up

Usual Care ITT, N=304

SMA ITT, N=494

SMA Attendee, N=374

SMA Engagement, N=294
